# *In Vitro* Efficacy of Curcumin-Loaded Amine-Functionalized Mesoporous Silica Nanoparticles against MCF-7 Breast Cancer Cells

**DOI:** 10.34172/apb.2023.035

**Published:** 2022-01-05

**Authors:** Zahra Mohebian, Mirzaagha Babazadeh, Nosratollah Zarghami

**Affiliations:** ^1^Department of Chemistry, Tabriz Branch, Islamic Azad University, Tabriz, Iran.; ^2^Department of Medical Biochemistry, Faculty of Medicine, Istanbul Aydin University, Istanbul, Turkey.

**Keywords:** Curcumin, Mesoporous silica nanoparticles, Drug delivery system, Breast cancer

## Abstract

***Purpose:*** Mesoporous silica nanoparticles (MSNs) have drawn substantial interest as drug nanocarriers for breast cancer therapy. Nevertheless, because of the hydrophilic surfaces, the loading of well-known hydrophobic polyphenol anticancer agent curcumin (Curc) into MSNs is usually very low.

***Methods:*** For this purpose, Curc molecules were loaded into amine-functionalized MSNs (MSNs-NH_2_ -Curc) and characterized using thermal gravimetric analysis (TGA), Fourier-transform infrared (FTIR), field emission scanning electron microscope (FE-SEM), transmission electron microscope (TEM), Brunauer-Emmett-Teller (BET). MTT assay and confocal microscopy, respectively, were used to determine the cytotoxicity and cellular uptake of the MSNs-NH_2_ - Curc in the MCF-7 breast cancer cells. Besides, the expression levels of apoptotic genes were evaluated via quantitative polymerase chain reaction (qPCR) and western blot.

***Results:*** It was revealed that MSNs-NH_2_ possessed high values of drug loading efficiency and exhibited slow and sustained drug release compared to bare MSNs. According to the MTT findings, while the MSNs-NH_2_ -Curc were nontoxic to the human non-tumorigenic MCF-10A cells at low concentrations, it could considerably decrease the viability of MCF-7 breast cancer cells compared to the free Curc in all concentrations after 24, 48 and 72 hours exposure times. A cellular uptake study using confocal fluorescence microscopy confirmed the higher cytotoxicity of MSNs-NH_2_ -Curc in MCF-7 cells. Further, it was found that the MSNs-NH_2_ -Curc could drastically affect the mRNA and protein levels of Bax, Bcl-2, caspase 3, caspase 9, and hTERT relative to the free Curc treatment.

***Conclusion:*** Taken together, these preliminary results suggest the amine-functionalized MSNs-based drug delivery platform as a promising alternative approach for Curc loading and safe breast cancer treatment.

## Introduction

 Breast cancer has been recognized as the main health burden globally among women. Incidence and mortality rates of breast cancer have been perceived to be rising over the past few years in many countries. Breast cancer current therapeutic approaches, including surgical removal, radiotherapy, hormone therapy, and chemotherapy, have shown some positive outcomes along with some disadvantages such as poor patient response, high risks of relapse, the emergence of drug resistance, and toxicity on normal cells.^1-3^

 Many reports have demonstrated phytochemicals, especially curcumin (Curc), as promising, safe and effective natural anticancer agents to enhance the efficiency of cancer therapy and minimize adverse reactions.^4,5^ The polyphenolic phytochemical Curc, isolated from turmeric rhizome, has been widely investigated for its anti-tumor effects. Several clinical trials reported the promising anticancer efficacy of Curc alone or combined with other standard chemotherapeutic drugs.^6,7^ Numerous investigations indicate that Curc impedes the growth of breast cancer cells through several signalling pathways such as affecting the expression of signalling proteins, including Wnt/β-catenin, mammalian target of rapamycin (mTOR), phosphatidylinositol-3-kinase (PI3K), protein kinase B (Akt), and Ras; induction of cell cycle arrest and p53-dependent apoptotic pathway; downregulation of some transcriptional factors, and inhibition of tumor angiogenesis.^8,9^

 Despite the corroborated anti-tumor activity of Curc, its weak solubility in water, poor bioavailability, and rapid metabolism are the major drawbacks for successful clinical applications of this dramatic phytochemical.^10^ Various approaches such as polymeric nanoparticles, silica nanoparticles, liposomes, niosomes, and metal or non-metal nanoparticles have been ever developed for encapsulation of Curc to improve its bioavailability.^11-14^

 Amongst these strategies, mesoporous silica nanoparticles (MSNs) have drawn substantial interest as drug nanocarriers because of their distinctive and excellent properties such as high surface area, well-ordered internal mesopores with high pore volume, tunable shape and size, straightforward surface functionalization, and excellent biocompatibility.^15,16^ Besides, MSNs increase the poor aqueous solubility of hydrophobic biomolecules and enhance their bioavailability.^17^ Moreover, the MSNs with diameters of 100-150 nm mostly accumulate at tumor sites due to the enhanced permeability and retention (EPR) effect.^18,19^ These characteristics have made MSNs ideal nanocarriers for cancer therapy. Nevertheless, because of the hydrophilic surfaces, the loading of hydrophobic anticancer molecules such as Curc onto MSNs is commonly very low.^20^ Modifying and functionalization of the surface of MSNs with desirable functional groups can provide numerous binding sites and thus large quantity of biomolecules is loaded onto the matrix and surface of MSNs.

 This study aims to design and synthesize amine-functionalized MSNs using (3-Aminopropyl) triethoxysilane (APTES) molecules to enhance the loading efficiency of a poorly water-soluble drug Curc, subsequently increasing its cytotoxicity and anticancer effects against MCF-7 breast cancer cells.

## Materials and Methods

###  Materials

 Curcumin, dimethyl sulfoxide (DMSO), (3,4,5-dimethyl thiazol-2-yl)-2,5 diphenyl tetrazolium bromide (MTT), 4′,6-diamidino-2-phenylindole (DAPI) and Glutaraldehyde were purchased from Sigma-Aldrich (St. Louis, MO, USA). Triton-X-100, triethanolamine (TEA), tetraethoxysilane (TEOS), cetyltrimethylammonium bromide (CTAB) and APTES were purchased from Merck (Darmstadt, Germany). Fetal bovine serum (FBS), RPMI 1640, penicillin G, streptomycin and Trypsin-EDTA were all provided from Gibco (Invitrogen, Paisley, UK). All other chemicals and reagents were mainly of analytic grade from commercial sources and were used without further purification.

### Preparation of MSNs and MSNs-NH_2_

 MSNs were prepared as depicted in earlier reports.^21^ 0.08 g of TEA and 2 g of N-cetyltrimethylammonium bromide (CTAB) were blended in 20 mL of deionized water, heated to 95°C, and stirred for 2 hours at 85°C. Afterwards, tetraethyl orthosilicate (TEOS, 1.5 mL) was added to the solution and stirred for an additional 2 hours before white precipitation occurred. To remove the remaining reactants, the obtained precipitate was gathered via centrifuging and washed with absolute ethanol. The MSNs were gained after vacuum drying for 24 hours at 55°C. Lastly, calcination at 600°C for 8 hours was used to extract the CTAB (surfactant template).

 Amine-functionalized MSNs (MSNs-NH_2_) were obtained by treating MSNs with APTES. Briefly, in a mixture containing 100 µL of APTES and 50 mL of ethanol, 50 mg of MSNs was stirred for 48 hours at 100°C. Next, the mixed solution was centrifuged, and MSNs-NH_2_ was gained after washing with distilled water.

###  Preparing and characterizing MSNs-NH_2_ -Curc

 MSNs-NH_2_-Curc was obtained according to the previously reported works.^22^ First, 500 mg of MSNs-NH_2 _and 20 mg of Curc were dispersed ultrasonically in 10 mL of acetone/ethanol (30/70, v/v) and placed under stirring at 37°C for 12 hours to generate MSNs-NH_2_-Curc. Then, the samples were centrifuged at 5000 rpm (5°C, 30 min), and the supernatants were gathered. The pellet was vacuum dried at a temperature of 55°C for 24 hours to evaporate the ethanol gradually. A UV-Vis spectroscopy (PerkinElmer Fremont, CA) was applied to quantify the Curc content in the collected supernatant at 425 nm with a standard curve of Curc. The drug encapsulation efficiency (EE%) and loading capacity (LC%) of Curc in the nanoparticles was indirectly calculated via the below equations.^23^


(1) $$Encapsulation\;efficiency\left(EE\%\right)=\frac{\left(Total\;amount\;of\;drug\;added-free\;non-entrapped\;drug\right)}{Total\;amount\;of\;drug\;added}\times 100$$

(2) $$Loading\;capacity\left(LC\%\right)=\frac{\left(Total\;amount\;of\;drug\;added-free\;non-entrapped\;drug\right)}{Total\;mass\;of\;nanoparticles}\times 100$$

Further, thermogravimetric analysis (TGA, Perkin Elmer, Fremont, CA) was used to calculate the amount of Curc encapsulated in the MSNs through the weight loss of airborne particles with a heating rate of 10°C min^-1^ from room temperature to 800°C.

 Field emission scanning electron microscope (FE-SEM) (MIRA3 TESCAN, Czech Republic) and transmission electron microscope (TEM) (Hitachi H-800, Japan) were utilized to observe the particle size and morphological features of the prepared MSNs. ImageJ (NIH, Bethesda, MD, USA) program was applied to determine the diameter and distribution of over 500 particles per FE-SEM sample. Further, a dynamic light scattering (DLS) Zetasizer Nano ZS (Malvern Instruments Ltd., Malvern, UK) was utilized to measure the mean particle size, polydispersity index (PDI), and zeta potential of the particles.

 The interaction between Curc molecules and synthesized MSNs was explored through a Fourier-transform infrared (FTIR) spectroscopy (IR330; Thermo Fisher Scientific, Waltham, MA). Also, the nitrogen adsorption-desorption isotherms were analyzed applying a physisorption analyzer (Micromeritics TriStar 3000, USA). The specific surface area of MSNs was measured based on the Brunauer–Emmett–Teller (BET) analysis, and Barrett–Joyner–Halenda (BJH) analysis was employed to assess the pore size distributions of MSNs from the desorption branches of isotherms.

###  In vitro drug release study

 The *in vitro* release of Curc from MSNs-Curc and MSNs-NH_2_-Curc was investigated via dialysis technique for 7 days. In brief, 2.0 mg of the nanoparticles dissolved in 1.0 mL of PBS (pH 7.4) were placed in a dialysis bag (MWCO = 3500), immersed into the 19 mL of PBS, and then incubated at 37°C in a thermostatic shaker. At particular time intervals, 1 mL of the PBS was replaced with 1 mL fresh PBS for capacity adjustment. The quantity of drug discharged in the withdrawal solutions was measured utilizing a UV-Vis spectrophotometer at an absorbance of 425 nm and a standard curve of Curc.

###  Cytotoxicity evaluation

 The cytotoxicity of free Curc, MSNs-NH_2_ and MSNs-NH_2_-Curc was investigated against MCF-7 breast cancer cells and MCF-10A non-tumorigenic breast cells after 24, 48 and 72 hours of incubation using MTT assay. First, the cells were seeded into 96-well plates (2 × 10^4^ cells/well) and incubated overnight at 37°C in a humidified atmosphere containing 5% CO_2_. The doubling time for each of the breast cell lines was obtained to be somewhat similar (MCF-10A, 25 hours; MCF7 and 29 hours). So, there was approximately the same number of cells at the end of the overnight incubation period. Then, the cells were exposed for 24, 48 and 72 hours to various concentrations of free Curc (0, 1, 5, 10, 15, and 20 µg/mL), MSNs-NH_2_ (0, 1.75, 8.74, 17.47, 26.2, and 34.95 µg/mL) and MSNs-NH_2_-Curc (0, 2.75, 13.74, 27.47, 41.2, and 54.95 µg/mL). The amounts of MSNs-NH_2_-Curc used in this study were equivalent to the free Curc concentrations used, considering the %LC of Curc. For the MTT assay, the media were substituted with 200 µL of MTT solution (0.5 mg/mL in PBS) and incubated for 4 hours at 37°C. Next, the media was substituted with 200 µL of pure DMSO and incubated for 20 minutes. To end, the absorbance was measured at the formazan maximum absorbance wavelengths (570 nm) applying a microplate reader (BioTek Power Wave XS). The relative cell viability was determined utilizing the below equation:


(3) $$Cell\;viability\left(\%\right)=A_{570}\left(sample\right)/A_{570}\left(control\right)\times 100$$

###  In vitro cellular uptake study 

 MCF-7 cells were seeded in a 24-well plate and incubated with free Curc and MSNs-NH_2_-Curc with IC_50_ values to assess the cellular uptake. After 24 hours, the cells were washed with Dulbecco’s phosphate-buffered saline (DPBS), fixed and permeabilized with 2.5% glutaraldehyde solution for 10 min and 0.1% Triton-X-100 for 15 minutes, respectively. Subsequently, the cancer cells were stained with 1 mg/mL of DAPI at 37°C for 10 minutes. A laser scanning confocal microscope (710, Carrel Zeiss, Jena, Germany) was applied for cell imaging.

###  Quantitative polymerase chain reaction (qPCR) 

 The qPCR technique was applied to investigate the influence of MSNs-NH_2_-Curc on the mRNA levels of apoptosis-associated genes. Briefly, the MCF-7 cells were exposed to the free Curc, MSNs-NH_2_ and MSNs-NH_2_-Curc with IC_50_ values for 72 hours. Then, the total RNA of the cells was isolated applying TRIzol® reagent (Invitrogen, NY, USA). A Nanodrop (NanoDrop Technologies Inc., Wilmington, DE, USA) was utilized to analyze the RNA integrity and purity. Afterwards, 1 μg of total RNA was applied to generate cDNA based on the manufacturer’s procedure with the RevertAid^TM^ First Strand cDNA Synthesis kit (Fermentas; Thermo Fisher Scientific, Inc., Pittsburgh, PA, USA). cDNA, specific primers, and SYBR Green PCR Master Mix (TaKaRa, Dalian, China) were applied to conduct qPCR assay through a Mic qPCR Cycler (BioMolecular Systems, Australia). Glyceraldehyde-3-phosphate dehydrogenase (GAPDH) gene was utilized as an internal control to compare the gene expression data, and the quantification of the samples was analyzed using the 2-ΔΔct method.

###  Western blot analysis

 MCF-7 cells treated with free Curc, MSNs-NH_2_ and MSNs-NH_2_-Curc for 72 hours were collected and lysed with RIPA buffer [10 mM Tris-HCl (pH 7.4), 150 mM NaCl, 1 mM EGTA, 0.3 mM PMSF, 0.2 mM sodium orthovanadate, 0.1% SDS, 1 mM EDTA, 1% NP-40, 10 mg/mL leupeptin, and 10 mg/mL aprotinin] and then, the protein concentration was measured using Bio-Rad Protein Assay kit (Bio-Rad, Hercules, CA, USA). The samples were subjected to 10% SDS-PAGE following heat denaturation at 95°C for 5 min. Subsequently, the target proteins in the gel were transferred onto polyvinylidene difluoride (PVDF) membranes (Bio-Rad Laboratories, Inc., Hercules, CA, USA), and the membranes were blocked with 5% BSA for 90 minutes at room temperature. The membranes were incubated with the following primary antibodies: Bcl-2 (dilution 1:1000, Santa Cruz, USA), Bax (dilution 1:5000, Santa Cruz, USA,), Caspase-3 (dilution 1:2000, Santa Cruz, USA), Caspase-9 (dilution 1:2000, Santa Cruz, USA), hTERT (1:1000, Santa Cruz, USA), and anti-GAPDH (1:250, Santa Cruz, USA) at 4°C overnight. After washing with phosphate-buffered saline with Tween20 (PBST), the blots were incubated with secondary antibody, anti-mouse IgG (1:5000, Santa Cruz USA), and the signals were revealed by enhanced chemiluminescence detection (Amersham Biosciences ECLTM).

###  Statistical analysis

 All values were expressed as mean ± standard error of at least three experiments and compared using one-way and two-way ANOVA tests using GraphPad Prism 8.0 (GraphPad Software, Inc., San Diego, CA). Values of *P* < 0.05 were considered as significant.

## Results and Discussion

###  Preparation and characterization of MSNs-NH_2_ and MSNs-NH_2_ - Curc

**Figure 1:**
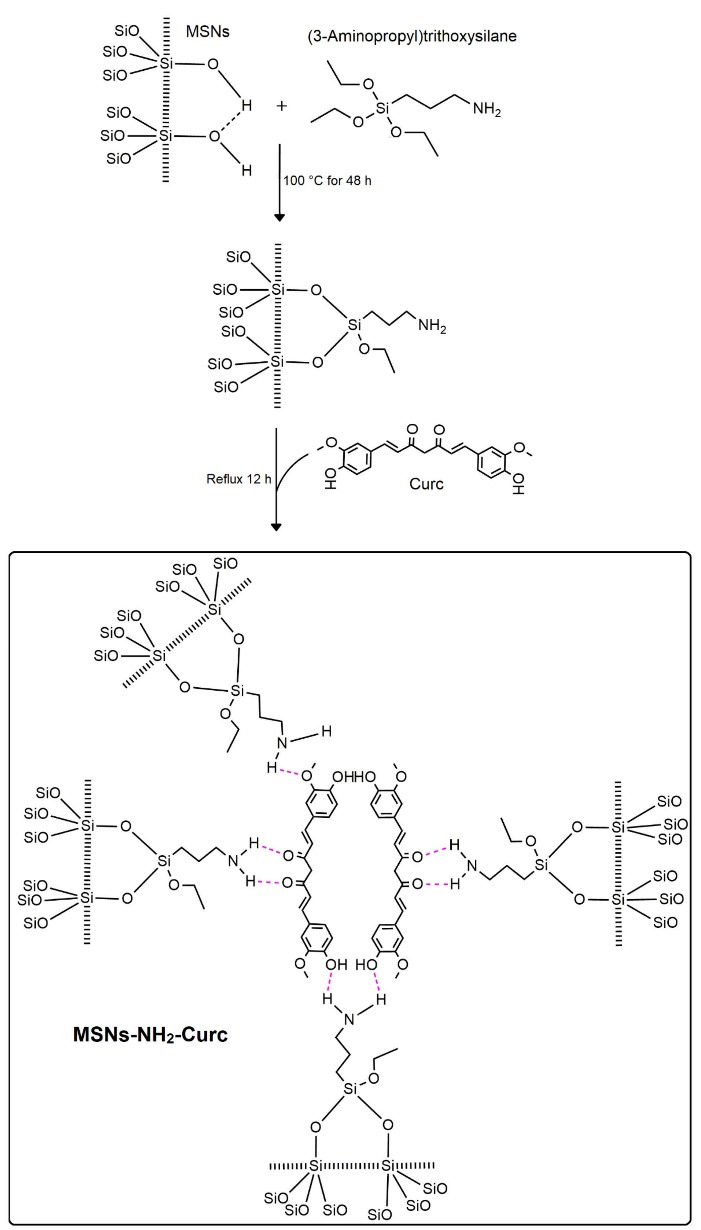
Schematic illustrating the amine-functionalization of MSNs and the interaction of Curc molecules with the MSNs-NH_2_.

 In this work, after amine-functionalization, the MSNs-NH_2_ were applied to load Curc Molecules (Figure 1). Several works showed that amine-functionalization onto MSNs using APTES could improve drug loading efficiency, generate a controlled release pattern, and enhance the bioavailability of Curc and other small drugs with poor water solubility.^24,25^

**Figure 2:**
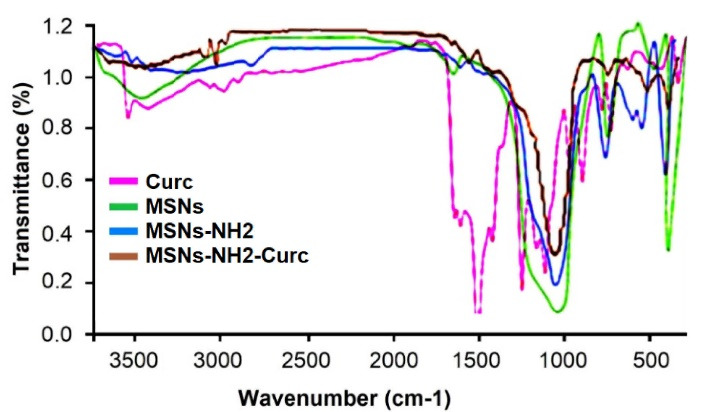
FTIR spectra of Curc, bare MSNs, MSNs-NH_2_, and MSNs-NH_2_-Curc.

 Successful functionalization of MSNs with APTES was confirmed by FTIR analysis (Figure 2). The FTIR spectra of MSNs, MSNs-NH_2_ and MSNs-NH_2_-Curc indicated similar peaks at 1100 cm^−1^ (Si-O-Si) and 950 cm^−1^ (Si-OH). The MSNs-NH_2_ displayed new peaks at 1565, 1630 1489, and 694 cm^−1^. The absorption peak at 694 cm^−1^ was ascribed to N-H as γ vibration in –NH_2_. Peaks at 1630 cm^−1^ and 1565 cm^−1^ were ascribed to N-H as δ vibration in -NH_2_. Peaks at 1489 cm^−1^ were attributed to C-H as -CH_2_- in APTES. Peaks around 2928 cm^−1^ attributed to C-H as σ vibration in -CH_2_- was increased after modification, further representing the successful amine functionalization of MSNs.^26^ The FTIR spectra of the MSNs-NH_2_-Curc possessed numerous peaks similar to free Curc. These peaks were detected for C–H stretching of aromatic rings at 3020 cm^−1^, C = O stretching at 1650 cm^−1^, C = O and C = C vibration at 1510 cm^−1^, and CH_3_ bending at 1300 cm^−1^. These spectra of MSNs-NH_2_-Curc displayed a new band at 3100 cm^−1^, proposing the creation of hydrogen bonding between phenolic -OH group of Curc and amine groups of MSNs-NH_2_.^27^

**Figure 3:**
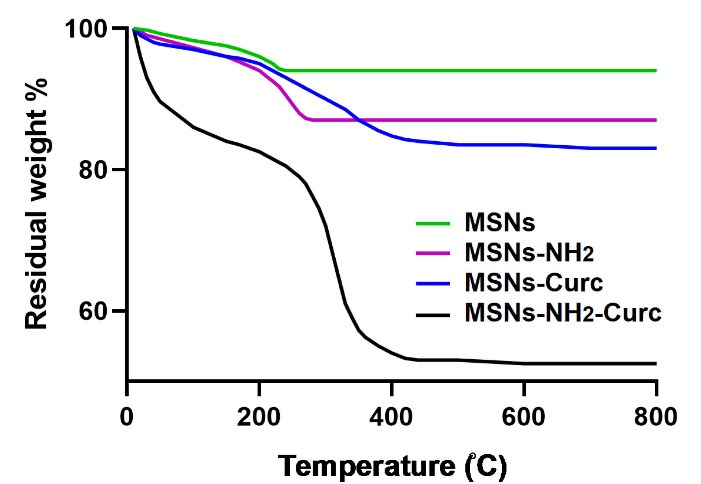
TGA thermograms of bare MSNs, MSNs-NH_2_, MSNs-Curc, and MSNs-NH_2_-Curc. The samples were placed in an Al pan and heated at a rate of 10°C/min from 25 to 800°C.

 UV-Vis spectroscopy and Eq. (1-2) were employed to determine drug encapsulation efficiency and loading capacity. LC% for MSNs-Curc and MSNs-NH_2_-Curc was found to be 9.1 and 36.8%, respectively. Besides, TGA was used to further determine the Curc loading capacity in the nanoparticles (Figure 3). According to the TGA data, the weight loss values of bare MSNs, MSNs-Curc, MSNs-NH_2_, and MSNs-NH_2_-Curc at 800°C were 6.5, 15.2, 11.2, and 47.2%, respectively. Therefore, LC% for MSNs-Curc and MSNs-NH_2_-Curc was calculated to be 8.7 and 36%, according to the data obtained from UV-Vis spectroscopy. The weight loss of MSNs-Curc and MSNs-NH_2_-Curc was due to the decomposition of amine groups and Curc molecules, respectively.

 It has been established that the amine-functionalization of MSNs considerably improve the loading capacity of Curc.^20^ Electrostatic interactions between the positively charged -NH_2_ groups existing on the surface of MSNs-NH_2_ and -OH and -C = O groups of Curc molecules play a crucial function in the enhanced loading capacity of these nanoparticles relative to that of the bare MSNs.^20,28^ Curc is hydrophobic in nature, comprising two vinyl and two phenyl groups. Thus, the hydrophobic interactions between the hydrophobic organic backbone of APTES molecules and Curc also increase the drug adsorption onto MSNs-NH_2_.

 Besides, FE-SEM, TEM, DLS, and nitrogen adsorption-desorption isotherm analyses were used for morphological and structural characterization of MSNs.

**Figure 4:**
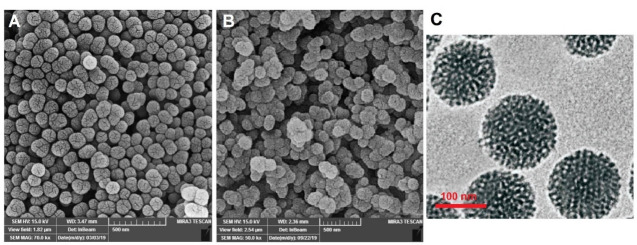
FE-SEM images of MSNs-NH_2_ (A, scale bar = 500 nm), MSNs-NH_2_-Curc (B, scale bar = 500 nm), and TEM image of MSNs-NH_2_ (C, scale bar = 100 nm). Distinguishable mesopores were observed on the surfaces of the MSNs-NH_2_ while encapsulating Curc into the MSNs-NH_2_ caused no detectable mesoporosity on their surface.

 As revealed in the FE-SEM images (Figure 4A and B), both MSNs displayed spherical forms with uniformity in distribution, and mean diameters of 89 ± 4 and 97 ± 6 for MSNs-NH_2_ and MSNs-NH_2_-Curc, respectively. Markedly, distinguishable mesopores were observed on the surfaces of the MSN-NH_2_ while encapsulating Curc into the MSNs-NH_2_ caused larger diameters with no detectable mesoporosity on their surface.

 In addition, the pore morphology of the MSNs was studied using TEM. From the TEM micrograph (Figure 4C), it could be observed that the pores of the MSNs were to some extent regular and have extended out from the center. These nanopores drastically enhance the specific surface area of MSNs and improve their ability for drug adsorption.

 DLS measurement was used to further assessment of the diameter and size distribution of nanoparticles. As presented in Table 1, MSNs-NH_2_ presented a mean diameter of 108 ± 9.7 nm with uniformity in size distribution and a PDI of 0.151. Through loading Curc molecules, the MSNs-NH_2_-Curc represented a larger size with an average hydrodynamic size of 128 ± 8.50 and a narrower particle size distribution (PDI = 0.123) due to the thickness of Curc around the MSNs and inside the pores too. The average diameters obtained via DLS was somewhat larger than that determined by FE-SEM because of the hydrate layer in the aqueous environment.

**Table 1:** Characterization of synthesized MSNs using DLS. The data are expressed as mean ± SD (n = 3)

**Groups**	**Particle size (nm)**	**Polydispersity index**	**Zeta potential (mV)**
Bare MSNs	102 ± 8.3	0.157	-22.6 ± 2.4
MSNs-NH_2_	110 ± 9.7	0.151	17.9 ± 2.3
MSN-NH_2_-Curc	132 ± 8.5	0.123	3.4 ± 1.5

 Also, the values of zeta potential were altered during the preparation of MSNs-NH_2_-Curc. As shown in Table 1, after amine-functionalization, the zeta potential of MSNs was raised from -22.6 ± 2.4 mV to 17.9 ± 2.3 mV. The alteration of zeta potential showed the effective synthesis of MSNs-NH_2_. After the loading with Curc, the zeta potential of MSNs-NH_2_ reduced to 3.4 ± 1.5 mV. The alterations in zeta potentials in each stage confirmed the successful amine-functionalization of MSNs.

 Mesoporous materials are characterized as organized structures with homogeneous pore diameters ranging between 2 nm and 20 nm, large surface area (ca. 1000 m^2^/g), high pore volume (ca. 1 cm^3^/g), and high density of silanol groups at their surface. These characteristics made MSMs an ideal platform for drug delivery systems requiring the high adsorption of biomolecules.^29^

 The pore diameter can be adjusted depending on the synthesis conditions and the surfactant utilized as a template. MSNs with a high pore size (up to 50 nm) are convenient for loading and delivering macromolecules such as nucleic acids, proteins, enzymes, and antibodies. In comparison, small molecules were efficiently adsorbed and loaded into the MSNs with small diameters (2-20 nm). The pore volume of conventional MSNs is a crucial element affecting the quantity of biomolecules that can be loaded, and the nanoparticles are well-recognized for hosting a large quantity of therapeutic molecules in the network of their cavities.

 Since drug loading is a superficial process, the large surface area of MSNs guarantees the great hosting ability of this kind of carrier, sometimes even exceeding 35 wt%.^30^

**Figure 5:**
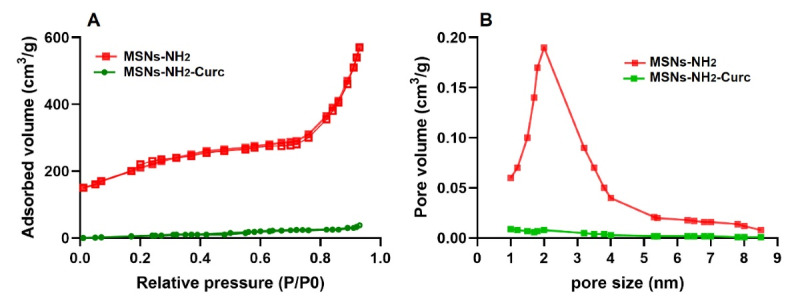
Mesoporous characteristics of MSNs. Nitrogen adsorption-desorption isotherms (A) and the corresponding pore size distribution (B) of MSN-NH_2_ and MSN-NH_2_-Curc.

 The BJH and BET analyses were applied to calculate the surface area, pore volume and pore size of MSNs, respectively (Figure 5). According to the IUPAC classification, MSNs presented a typical irreversible type IV isotherm corresponding to mesoporous material. It was shown that unloaded MSNs-NH_2_ has BET surface area, pore volume and pore size of 750 ± 17 m^2^/g, 0.92 ± 0.7 cm^3^/g, and 3.73 ± 0.6 nm, respectively. After encapsulating Curc, the surface area, pore volume and pore size of MSNs-NH_2_-Curc were diminished to 125.2 ± 3.4 m^2^/g, 0.27 ± 0.25 m^3^/g, and 1.33 ± 0.3 nm, respectively. Together with the FTIR, these results confirm that Curc has been successfully adsorbed into the MSNs-NH_2_ porous structure.

 The findings of this experiment are in accordance with previous reports in which MSNs-NH_2 _was used to load Curc molecules,^20,22,31^ suggesting the potential application of these mesoporous materials as efficient carriers for storing more quantities of small hydrophobic therapeutic molecules.

###  In vitro release of Curc

 Ideal drug delivery carriers must not only display high drug loading efficiencies and capacities but also must liberate the biomolecules in a controlled manner to create a sustained drug delivery platform. In this work, UV−Vis spectrophotometry was used to determine the *in vitro* release profile of Curc form MSNs-Curc and MSNs-NH_2_-Curc for 7 days at 37°C. Two methods of UV-Vis spectrophotometry and HPLC are mainly used for measuring drug entrapped in nanocarriers and the quantity of drug released. Rossi et al proposed these two methods as alternatives for the quantitation of Capreomycin in liposomal formulations.^32^ They showed that both methods were valid alternatives for quantitative drug analysis, even though the UV-Vis spectrophotometric method presented less accurate than the HPLC in reversed-phase mode. In fact, the spectrophotometric analyses were inexpensive, easier and required a shorter time for experiments. However, it has several limitations, including low sensitivity and selectivity. Several studies reported UV-Vis spectrophotometry as an acceptable method for quantitative drug analysis in less complex solutions such as those adopted *in vitro* release kinetics studies from the drug-loaded nanoparticles and quality control.^33,34^

**Figure 6:**
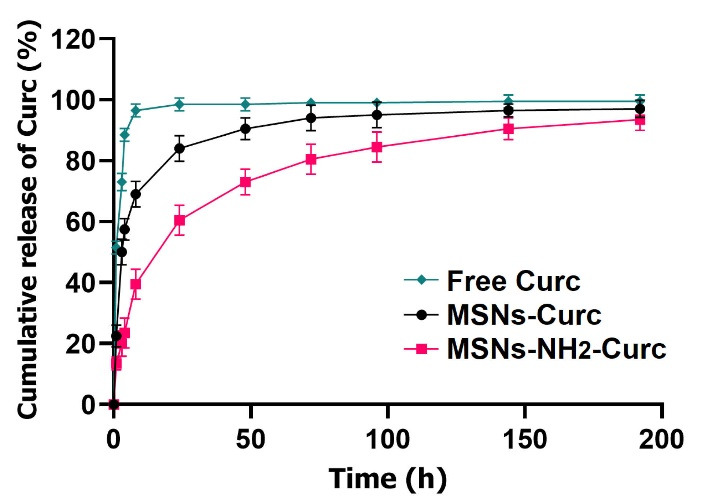
Cumulative release of Curc from MSNs-Curc and MSN-NH_2_-Curc, and free Curc into PBS (pH 7.4) at certain intervals of time, showing variations on the release of Curc *in vitro*. MSN-NH_2_-Curc exhibited a controlled drug release profile in which a slightly fast initial release in the first few hours was followed by a sustained and slower rate release over 7 days, and about 60%, 73% and 82% of Curc was released after 24, 48 and 72 h. Triplicate data and values are expressed in mean ± SD (n = 3).

 According to the drug release profiles (Figure 6) measured by UV-Vis spectrophotometry, a burst release of Curc from MSNs-Curc was observed in the first hours of incubation time so that over 85% of the drug was discharged within 24 hours. In contrast, MSNs-NH_2_-Curc exhibited a controlled drug release profile in which a slightly fast initial release in the first few hours was followed by a sustained and slower rate release over 7 days and about 60, 73 and 82% of Curc was released after 24, 48 and 72 hours, respectively.

 The fraction of drug, released within a few hours from MSNs-NH_2_-Curc, is probably related to the Curc molecules adsorbed on the nanoparticle surfaces, and the subsequent gradual discharge might happen because of the desorption of Curc molecules electrostatically interacted with the -NH_2_ groups of the APTES molecules present in the pores of MSNs.

 In MSNs-Curc, the hydrogen bonds between the silanol group of bare MSNs and -OH groups of Curc are not strong enough to hold the Curc inside mesopores, and therefore the release kinetics are the highest.^35^ In contrast, the strong electrostatic interactions between the -NH_2 _groups of the APTES molecules present on the surface of MSNs with -OH and -C = O groups of Curc are more effective to hold Curc, leading to slow-release kinetics.^23^ Besides, the hydrophobic interactions between the hydrophobic organic backbone of APTES molecules and Curc might contribute to the delayed release of the drug.

 These findings are exactly parallel with several works showing that amine-functionalization of MSNs could improve loading efficiency, generate a controlled release pattern, and enhance the bioavailability of Curc and other small drugs with poor water solubility.^20,36,37^

 In addition to the surface chemistry, the pore size of MSNs has a crucial function in the drug discharge rate as the drug liberation is principally controlled by diffusion. Jia et al developed paclitaxel-MSNs with pore diameters from 3 to 10 nm. Drug release analysis displayed that the liberation rate reduced when the pore diameters altered from 10 to 3 nm, which may be attributed to the less opportunity of loaded paclitaxel for escaping and diffusing from the relatively small pores into the release medium. The influence of pore size of MSNs on the drug discharge profile was further shown in the celecoxib-loaded MSNs.^38^ It has been shown that the discharge rate of celecoxib from MSNs enhanced with the rise of the pore diameter from 3.7 to 16.0 nm.

###  In vitro cytotoxicity of MSNs-NH_2_ -Curc

**Figure 7:**
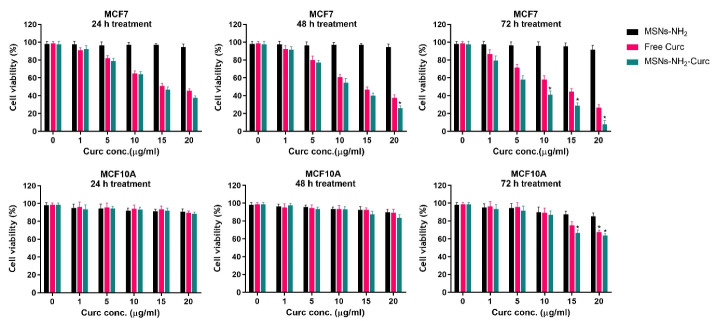
*In vitro* cytotoxicity of different concentrations of free Curc (0-20 µg/mL) and MSNs with drug content equal to the applied free Curc concentrations (0, 2.75, 13.74, 27.47, 41.2, and 54.95 µg/mL) on MCF-7 breast cancer cells and MCF-10A non-tumorigenic breast cells after 24, 48 and 72 h incubation time using MTT assay. MSN-NH_2_-Curc considerably decreased the viability of MCF-7 cells compared to the free Curc in a dose- and time-dependent manner. Besides, free Curc and MSN-NH_2_-Curc were nontoxic to the MCF-10A cells at low concentrations. *P < 0.05 vs untreated cells were considered signiﬁcant. The data are expressed as mean ± SD (n = 3).

 MCF-7 and MCF-10A cells were exposed to free Curc and MSNs with Curc content equal to the applied free Curc concentrations to assess the cytotoxicity. Then, cell viability was investigated via MTT assay after 24, 48 and 72 hours exposure times. As shown in Figure 7, both free Curc and MSNs-NH_2_-Curc substantially reduced the viability of the MCF-7 cells in a time- and dose-dependent manner after 24, 48 and 72 hours of treatment. It was found that MSNs-NH_2_-Curc significantly affects the cancer cells’ viability than free Curc (*P* < 0.05).

 In a similar trend, IC_50 _values were calculated to quantitate the amount of drug required to kill 50% of cancer cells. The obtained IC_50_ values for free Curc and MSNs-NH_2_-Curc in MCF-7 after 24, 48 and 72 hours exposure times were presented in Table 2. The data revealed that the IC_50_ values of MSNs-NH_2_-Curc were lower than free Curc, indicating the superior performance of MSNs-NH_2_-Curc.

**Table 2:** IC_50_ values for free Curc and MSNs-NH_2_-Curc against MCF-7 breast cancer cells

**Exposure time (h)**	**Free Curc (µg/mL)**	**MSNs-NH**_2_**-Curc (µg/mL)**
24	16.79	14.09
48	13.80	11.02
72	11.07	5.72

 Parallel to these findings, the cytotoxicity of Curc-loaded MSNs were reported in various cells such as human hepatocellular carcinoma HepG2 cells, A549 lung cancer cells, SKOV3 ovarian carcinoma cells, human melanoma cell line A375, human cervical adenocarcinoma HeLa cells, human colon carcinoma cell line HT-29, MKN-28 human gastric adenocarcinoma cells, and MDA-MB-231 breast cancer cells.^24,25,39,40^ These reports suggested that the increased cytotoxicity of Curc-loaded MSNs on different cancer cells may be due to the high intracellular concentration and controlled discharge of the Curc. It has been proven that free drug molecules simply diffuse via the cellular membrane. In contrast, the nanoparticulate drug carriers apply specific cellular uptake pathways and discharge the drug molecules in a controlled manner.

 The MTT findings on MCF-10A revealed that free Curc and MSNs-NH_2_-Curc were nontoxic to the healthy cells at low concentrations. However, with raising the concentration of the free Curc and MSNs-NH_2_-Curc (more than 15 µg/mL), the cytotoxic effects toward MCF-10A cells were significantly increased after 72 hours exposure time. Besides, the results indicated that the viability of the normal cells was not affected in the presence of the drug-free MSNs-NH_2_, representing their biocompatible nature. The biocompatibility of MSNs has been well-demonstrated in several normal cells such as MCF-10A, NIH-3T3 and CHO.^24,41,42^

###  Cellular uptake of MSNs-NH_2_ -Curc 

**Figure 8:**
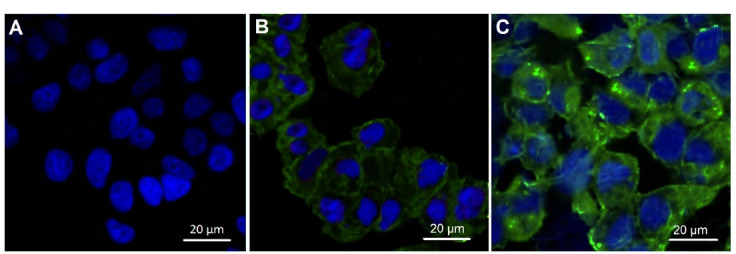
Confocal microscopy images of cellular uptake in untreated MCF-7 cells (A), and the cells treated with free Curc (B) and MSN-NH_2_-Curc (C) after 24 h incubation time (cell nuclei staining with DAPI-blue light, Curc-green light). Strong green fluorescence intensity was detected for MSN-NH_2_-Curc, demonstrating their high cellular uptake.

 To explore the reason for the cytotoxicity in cancer cells, the cellular uptake investigation was performed in MCF-7 breast cancer cells, and the distribution of MSNs-NH_2_-Curc inside the cells was detected on fluorescent images obtained via laser scanning confocal microscopy once Curc was excited at a wavelength of 488 nm. As shown in Figure 8, the distinct difference in cellular uptake of the MSNs-NH_2_-Curc and free Curc via the fluorescence intensity was clearly obtained. Poor green fluorescence intensity was visualized for the free Curc treatment, demonstrating the low cellular uptake of free Curc by the MCF-7 cells. On the other hand, strong green fluorescence intensity was detected for MSNs-NH_2_-Curc, indicating the high cellular uptake of MSNs-NH_2_-Curc, which may be due to the increased membrane permeability in the cancer cells. MSNs-NH_2_-Curc appear to be situated in the cytoplasm of the cancer cells in monolayer culture. It has been shown that amine-functionalized MSNs (hydrophilic and positively charged), compared to pristine MSNs (hydrophilic and negatively charged) and methyl-functionalized MSNs (hydrophobic and negatively charged), exhibit higher cellular uptake and anticancer activity owing to their electrostatic interaction with cancer cells.^36^

###  Gene expression analysis

 Curc can trigger apoptosis induction in most breast cancer cells by inducing cell membrane permeability, swelling and loss of membrane potential.^43^ Cell apoptosis via mitochondrial-dependent pathway triggered by Curc leads to the discharge of cytochrome C and triggering a caspase-9-caspase-3 cascade. Afterwards, cleaving poly (ADP-ribose) polymerase (PARP) by the ‘effector’ caspases-3 causes DNA fragmentation and eventually cell demolishing and apoptosis.^13,44^ Also, the upregulation of Bax and Bad and the downregulation of Bcl-XL and Bcl-2 can contribute to induce apoptotic pathways by Curc in breast cancer cells. Besides, Curc affects telomerase in various cancer cells. One of the hallmarks of cancer cells is their limitless self-renewal ability gained by telomere maintenance via telomerase reactivation. Transcriptional regulation of the catalytic subunit of telomerase, human telomerase reverse transcriptase (hTERT), is considered critical in telomerase reactivation in various cancer cells.^45-47^ Numerous reports proved that Curc could inhibit telomerase activity and hTERT expression in different tumors, especially breast cancer cells, in a dose- and time-dependent behavior.^48,49^

**Figure 9:**
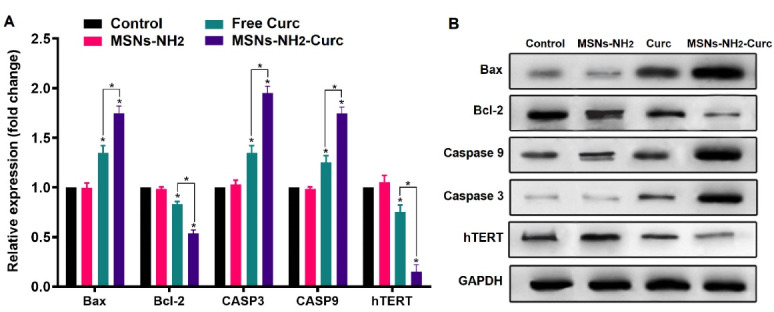
mRNA (A) and protein (B) levels of Bax, Bcl-2, caspase-3, caspase-9 and hTERT compared to GAPDH in MCF-7 breast cancer cells treated with free Curc, MSNs-NH_2_ and MSN-NH_2_-Curc after 72 h incubation time. MSN-NH_2_-Curc substantially altered the expression levels of Bax, caspase-3, caspase-9, Bcl-2, and hTERT. *P < 0.05 vs control was considered signiﬁcant. The data are expressed as mean ± SD (n = 3).

 Based on the qPCR results (Figure 9), it was revealed that both free Curc and MSNs-NH_2_-Curc significantly altered the mRNA expression levels of apoptotic genes and hTERT relative to the control group. Compared to free Curc, MSNs-NH_2_-Curc showed a substantial up-regulation in the Bax, caspase-3, and caspase-9 expression levels. Besides, down-regulation of anti-apoptotic gene Bcl-2 and hTERT was more noticeable in the cells treated with MSNs-NH^2^-Curc.

 Levels of apoptosis-related proteins, and hTERT, expressed in MCF-7 cells were also evaluated through western blot analyses. In accordance with the qPCR results, it was found that a 72-hour incubation with MSNs-NH_2_-Curc considerably increased the protein levels of caspase-3, and -9, and Bax and decreased the protein levels of Bcl-2 and hTERT compared to the free Curc, indicating that MSNs-NH_2_-Curc efficiently suppress MCF-7 cell proliferation through apoptosis via the mitochondria-related apoptotic pathway. The greatly enhanced pro-apoptosis effect is most probably resulted from the targeted delivery of anticancer agent into cells, which increases intracellular drug concentration and bypasses the transmembrane effluxing mechanism.

 The results are consistent with previous studies showing that nanocurcumin inhibits the proliferation of breast cancer cells by activating apoptosis better than free Curc.^24,50^

 The aqueous solubility and consequent cellular uptake of the hydrophobic free Curc molecules into the cells is low, so small amounts of Curc in the cells exposed to the free Curc were able to enter the cells, while cells have a high affinity for hydrophilic MSNs; therefore, large amounts of Curc molecules were able to enter the cells through uptaken MSNs-NH_2_-Curc. Thus, the intracellular concentration of Curc in the cells treated with MSNs-NH_2_-Curc is higher than those exposed to free Curc. Besides, the relatively slow and sustained release of drug molecules from MSNs-NH_2_-Curc can also lead to a steep rise in the anti-tumour performance of Curc. Consequently, it can be expected that MSNs-NH_2_-Curc exhibit a significant impact on induction of apoptosis compared to the free Curc treatments after 72 hours incubation time.

## Conclusion

 In summary, amine-functionalized MSNs (MSNs-NH_2_) were designed and prepared for enhancing the loading capacity of Curc, a poorly water-soluble therapeutic molecule, improving the drug bioavailability, cellular uptake and eventually increasing its anticancer efficiency. Because of the electrostatic interactions between Curc molecules and functional groups existing on the surface of MSNs-NH_2_, higher values of drug loading capacity and controlled drug release profile were obtained. The MSNs-NH_2_-Curc could be efficiently taken up by the MCF-7 breast cancer cells and specifically released Curc intracellularly in a controlled manner, which led to considerable cytotoxicity against MCF-7 cells and lack of cytotoxic effect on MCF-10A human breast epithelial cells. Besides, apoptotic genes’ mRNA and protein levels and hTERT could be significantly altered through exposure to the MSNs-NH_2_-Curc. These preliminary *in vitro* findings revealed that MSNs-NH_2_ might be a promising nanocarrier to improve the bioavailability and anticancer efficacy of Curc molecules against breast cancer cells.

## Acknowledgments

 The authors thank the Department of Clinical Biochemistry and Laboratory Medicine, Faculty of Medicine, Tabriz University of Medical Sciences for all support provided.

## Competing Interests

 The authors declare no conflicts of interest.

## Ethical Approval

 Not applicable.
